# Supplementary material for: Compact realization of all-attosecond pump-probe spectroscopy
Source: Sci Adv. 2024 Feb 21;10(8):eadk9605. doi: 10.1126/sciadv.adk9605 (PMC10881040; doi:10.1126/sciadv.adk9605)
Supplement: Supplementary file 1 — Sections S1 to S4 Figs. S1 and S2 [file sciadv.adk9605_sm.pdf]

Science Advances
AAAS

Supplementary Materials for

Compact realization of all-attosecond pump-probe spectroscopy

Martin Kretschmar *et al.*

Corresponding author: Bernd Schütte, bernd.schuette@mbi-berlin.de

Sci. Adv. **10**, eadk9605 (2024)
DOI: 10.1126/sciadv.adk9605

This PDF file includes:

Sections S1 to S4
Figs. S1 and S2

I. XUV SPECTRA USED IN APAPS

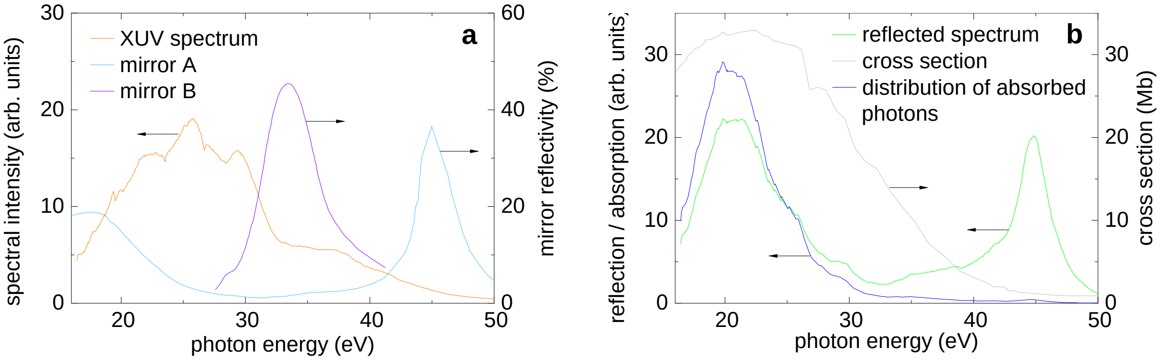

FIG. 1: XUV spectra used for APAPS. a, XUV spectrum for HHG in Kr (orange curve) and calculated reflectivities from mirror A (violet curve) and mirror B (cyan curve). **b**, Reflected spectrum from mirror A (green curve), photoionization cross section of Ar obtained from Ref. [33] (gray curve) and the predicted spectral distribution of the absorbed photons (blue curve).

In Fig. 1**a**, the XUV spectrum obtained for HHG in Kr (orange curve) is shown together with calculated reflectivity curves of mirrors A (violet curve) and B (cyan curve). Mirror A has two reflectivity peaks at about 18 eV and 45 eV. To identify which part of the XUV spectrum is most relevant in the experiment, the green curve in Fig. 1**b** shows the XUV spectrum after reflection from mirror A. In addition, the ionization cross section for neutral Ar is shown as gray curve. The blue curve depicts the predicted spectral distribution of absorbed photons, showing that the latter is dominated by photons with energies around 20 eV.

II. SPATIAL CROSS-CORRELATION

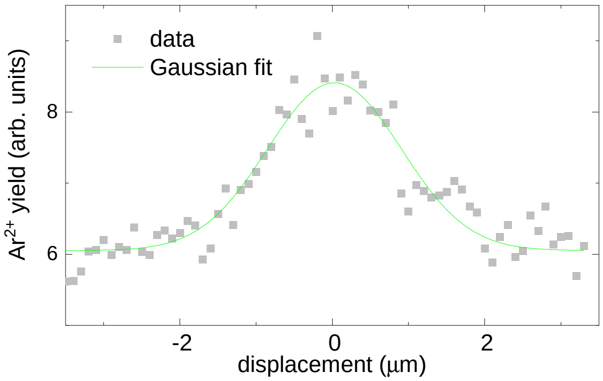

FIG. 2: Spatial cross-correlation. Ar^{2+} ion yield (gray data points) as a function of the displacement of the foci of the pump beam and the probe beams in the two-color APAPS experiment. Here the probe pulse arrived 20 fs after the pump pulse. The Gaussian fit (green curve) gives a value of 1.75 μm. Assuming that the focus sizes of the two beams are similar, the XUV beam waist radius is estimated as 1.2 μm.

To estimate the XUV focus size, we have performed a measurement in which the probe pulse arrived 20 fs after the pump pulse, and the spatial overlap was varied in the horizontal direction using a closed-loop mirror mount. As a result, the XUV fluence is changed, thereby altering the efficiency of sequential two-photon ionization [18]. The result is shown in Fig. 2 (gray data points), and the Gaussian fit (green curve) gives a radius of 1.7 μm for the cross-correlation. Assuming that the XUV focus sizes of the pump and the probe beams are similar, we estimate the XUV beam waist radius in horizontal direction to be 1.2 μm. Assuming further that the XUV beam waist radius

is two times larger in the vertical direction due to the splitting of the beam, the beam waist radius in the vertical direction is estimated as 2.4 μm. These values were used for the estimation of the XUV peak intensities.

III. DERIVATION OF FORMULA USED FOR ESTIMATING SIGNAL LEVELS IN APAPS

In order to assess the signal levels in APAPS experiments, we consider an atom at position (r, z) where r corresponds to the radial coordinate and z corresponds to the coordinate in the propagation direction. The probability that this atom excited respectively ionized by the pump and the probe is given by the product of the photon fluence $F_{pump,probe}(r, z)$ and the cross section σ of the process. For a Gaussian beam the photon fluence is given by

$$\begin{aligned} F_{\text{pump/probe}}(r,z) &= \frac{I_0}{\nu_{\text{pump/probe}}} \times \frac{1}{1+z^2/z^2_{R,\text{pump/probe}}} e^{-2r^2/w^2_{z,\text{pump/probe}}} \\ I_{0,\text{pump/probe}} &= N_{\text{pump/probe}} \nu_{\text{pump/probe}} \frac{2}{\pi w^2_{0,\text{pump/probe}}} \\ w^2_{z,\text{pump/probe}} &= w^2_{0,\text{pump/probe}}(1+z^2/z^2_{R,\text{pump/probe}}), \end{aligned} \tag{1}$$

where $I_{0,\text{pump/probe}}$ is the peak intensity of the pump / probe pulse, $\nu_{\text{pump/probe}}$ is the corresponding photon energy, $z_{R,\text{pump/probe}}$ is the Rayleigh length, $N_{\text{pump/probe}}$ is the number of photons, and $w_{z,\text{pump/probe}}$ is the beam radius at a distance z from the focus. Assuming that the pump pulse follows the probe pulse without temporal overlap, the probability $P(r, z)$ for two-photon double ionization is

$$P_{\text{pump+probe}}(r,z) = P_{\text{pump}}(r,z)P_{\text{probe}}(r,z) = \frac{2N_{\text{pump}}\sigma_{\text{pump}}}{\pi w^2_{z,\text{pump}}} \frac{2N_{\text{probe}}\sigma_{\text{probe}}}{\pi w^2_{z,\text{probe}}} e^{-2r^2(1/w^2_{z,\text{pump}}+1/w^2_{z,\text{probe}})}. \tag{2}$$

The number of two-photon events is given by the volume integral over the product of the atomic density and the position-dependent probability:

$$N_{\text{event}} = \int_0^r 2\pi r dr \int_{-\infty}^{\infty} dz\, n(r,z) P_{\text{pump+probe}}(r,z) \tag{3}$$

Assuming that $z_R \ll d$ (d being the diameter of the gas beam), we find

$$N_{event} = \frac{2n_0 N_{\text{pump}}\sigma_{\text{pump}} N_{\text{probe}}\sigma_{\text{probe}}}{\pi} \int_{-\infty}^{\infty} \frac{dz}{w^2_{z,\text{pump}} + w^2_{z,\text{probe}}}. \tag{4}$$

After evaluating this integral for two different cases, where either $\lambda_1 \gg \lambda_2$ or $\lambda_1 = \lambda_2$, we end up with the formula given in the main manuscript:

$$N_{\text{event}} = c \times \frac{2\pi n_0 N_{\text{pump}}\sigma_{\text{pump}} N_{\text{probe}}\sigma_{\text{probe}}}{\lambda_{\text{pump}}}, \tag{5}$$

where $c = 1$ if $\lambda_{\text{pump}} \gg \lambda_{\text{probe}}$ and $c = 0.5$ if $\lambda_{\text{pump}} = \lambda_{\text{probe}}$.

We note that this derivation was made for two collinear beams. In the experiment two non-collinear XUV beams were used, for which we estimate that the number of two-photon events is reduced by a factor of about 3.

IV. FITTING OF APAPS SIGNALS

The two-photon cross-correlation (CC) and auto-correlation (AC) signals were modeled assuming that both processes are driven by a short attosecond pulse train consisting of one central attosecond pulses with duration τ and one equally large pre- and post-pulse (with the same pulse duration). The CC and AC signals were modeled by evaluating the delay-dependent time-integral of the intensity distribution of the two interacting XUV fields E_1 and E_2. In the expression for the auto-correlation, it is assumed that a direct two-photon transition accounts for the delay-dependent

Ar^{2+} signal S_{AC}, which is obtained by evaluating

$$S_{\mathrm{AC}}(\tau) = \int_{-\infty}^{\infty} I(t,\tau)^2 dt. \tag{6}$$

Here $I(t,\tau)$ is the intensity and τ is the temporal delay between the contributing fields. We neglect fringe-resolved contributions in the field-interaction, reducing the delay-dependent intensity of the two synthesized fields to $I(t,\tau) = |E_1(t)+E_2(t+\tau)|^2 \approx |E_1(t)|^2+|E_2(t+\tau)|^2$, which was shown to give reasonable predictions for focal-volume averaged signals [18].

For the estimation of the two-color cross-correlation signal of Ar^{2+}, it is assumed that a purely sequential pathway accounts for the delay-dependent signal S_{CC}, evaluated by

$$S_{\mathrm{CC}}(\tau) = \int_{-\infty}^{\infty} |E_2|^2 \int_{-\infty}^{t} I(t',\tau)dt'd\tau. \tag{7}$$

The equation expresses that the cross-correlation signal that is generated from an intermediate population of singly-charged ions ($P(t,\tau) \approx \int_{-\infty}^{t} I(t',\tau)dt'$) can only be depleted by the second XUV pulse.

Based on Eqns. 6 and 7, a fitting procedure was performed to obtain an attosecond pulse that is consistent with both the cross-correlation and the auto-correlation experimental data. The fitting approach uses global parameters of a generalized attosecond pulse for the optimization, where each burst has a width of τ_{FWHM} with a temporal separation τ_{sep} between different attosecond bursts and a relative intensity $I_{\mathrm{pre-post}}$ of the pre- and post-pulses. An additional consideration of background signals in the experimental traces yields a good fit to the experimental datasets, as shown in Fig. 3 and Fig. 4 of the main manuscript. The obtained values from the fit are $\tau_{\mathrm{FWHM}} = 240$ as, $\tau_{\mathrm{sep}} = 800$ as and $I_{\mathrm{pre/post}} = (16 \pm 2)$ %.
